# The Preschool Physical Literacy Assessment Tool: Testing a New Physical Literacy Tool for the Early Years

**DOI:** 10.3389/fped.2018.00138

**Published:** 2018-06-07

**Authors:** John Cairney, Heather J. Clark, Maeghan E. James, Drew Mitchell, Dean A. Dudley, Dean Kriellaars

**Affiliations:** ^1^Infant and Child Health Lab, Faculty of Kinesiology and Physical Education, University of Toronto, Toronto, ON, Canada; ^2^Physical Literacy Research Group: Sport for Life Society, Vancouver, BC, Canada; ^3^Faculty of Human Sciences, Macquarie University, Sydney, NSW, Australia; ^4^Faculty of Health Sciences, College of Rehabilitation Sciences, University of Manitoba, Winnipeg, MB, Canada

**Keywords:** physical literacy, preschool, physical activity promotion, psychometric properties, psychometric, validity, reliability

## Abstract

**Background:** Physical literacy is essential to physical activity across the lifespan. While there is an emerging body of research on physical literacy in school-aged children, the preschool years have largely been ignored. We tested the psychometric properties of the new tool, the Preschool Physical Literacy Assessment Tool (Pre-PLAy) designed to address this gap.

**Methods:** We recruted 78 children (aged 19–49 months) across 5 childcare centers in Hamilton, Ontario. Two Early Childhood Educators (ECE) completed the Pre-PLAy for each child at two points in time to assess inter-rater reliability and test-retest reliability. We assessed the agreement between the Pre-PLAy tool with gross motor skills and the ability of the PPLAy to predict physical activity.

**Results:** Results indicated Pre-PLAy is related to gross motor skills and predictive of physical activity for females, but not males. Inter-rater and intra-rater reliability was at least adequate for all but the coordinated movements items and scale for females, but ECEs showed poor agreement for males.

**Conclusions:** These results suggest initial support for the Pre-PLAy tool as a measure of physical literacy during the early years. However, some modification to the items and training are required to address the gender-specific effects found in this sample.

## Introduction

Physical literacy (PL) encompasses the knowledge, skills, motivation, and feelings related to physical activity (PA) and movement (1). There is a growing interest regarding the definition and utility of PL by academics and professional organizations [e.g., (1–3)]. Despite this interest in PL, the preschool years have largely been ignored. This is an important gap as PL has been proposed as foundational to engagement in PA (2) and PA behaviors are established early in life (4). Therefore, supporting PL in the early years has the potential to increase PA. Understanding and supporting PL in the early years is important considering the concern regarding the low activity levels among young children (5) and the prevalence of obesity (6).

A PL assessment instrument for the preschool years is needed to address the PL research gap at this age. Existing PL measures were developed for school-aged children (3, 7). Without a PL tool for the early years, researchers have relied on measures of motor skill to assess PL. This approach fails to assess the multi-dimensional and lifelong aspects of PL and can contribute to confusion regarding the construct (8, 9). We developed the Preschool Physical Literacy Assessment Tool (Pre-PLAy) to be completed by Early Childhood Educators (ECEs) for children ages 18 months to kindergarten entry (about age 4). Pre-PLAy was designed to measure PL that assess the following domains of PL, consistent with the literature: Movement competences, coordinated movements, motivation, and enjoyment (2, 10). Knowledge of the importance of PA has also been identified as a domain of PL (1, 2). However, we did not include it in Pre-PLAy as we did not consider it developmentally appropriate for preschool children. Preschool children will neither have acquired nor be able to demonstrate knowledge regarding the importance of PA.

All items are scored based on ECE observations of the child across structured, free play, and self-care (e.g., dressing) activities. The movement competences items measure competence in specific skill domains (e.g., object control skills) and coordinated movements items assess coordinated executions of multiple skills simultaneously (e.g., running and striking). In motivation and enjoyment domains ECEs are instructed to complete items related to motivation and enjoyment based on behaviors, reactivity, and enjoyment while active. This approach is necessary because children at this age are not able to reliably self-report their perceived competence in specific areas (11).

The purpose of the study was to assess the reliability and validity of Pre-PLAy. To assess validity we examined the agreement between Pre-PLAy with a standardized test of gross motor skills and whether the Pre-PLAy predicted objectively measured PA. We expected a moderate correlation between the total Pre-PLAy and gross motor skills given that PL includes movement competences, but is also broader than movement competencies. For the subscales, we expect a stronger association between gross motor skills with the movement competencies and coordinated movements scales than motivation. Gross motor skills are conceptually related to movement competencies, though they are distinct (12).

We also hypothesized that Pre-PLAy would moderately predict PA. We posit a moderate relationship as the conceptual work of PL has positioned PL as foundational to PA [e.g., (2, 9, 13)], but previous research has reported low correlations between motor skill and PA during this developmental period (14). We anticipate the inclusion of motivation and enjoyment in the PL construct, will demonstrate a stronger relationship than motor skill alone has shown.

We will also examine gender differences, considering past research has demonstrated sex differences in PL and related constructs. For example, Longmuir (3) reported gender differences in the physical competence domain of PL measured by the Canadian Assessment of Physical Literacy in a sample of school aged children. Sex differences were also found in overall PL and other subdomains, but only for children older than age 10. Other research has reported gender differences in motivation and enjoyment of PA [e.g., (15)].

## Materials and methods

### Sample

We recruited 78 children and 26 female ECEs across 5 childcare centers in Hamilton, Ontario. Children who were under 18 months, had a diagnosis of developmental delay (e.g., autism, cerebral palsy), attended the center for <2 full days each week, or were leaving the class or center before the study period finished were excluded from the study. Research staff also attended the center during pick-up time to speak with parents, answer any questions, and obtain consent for eligible participants.

### Measures

#### Physical literacy

**Figure 1:**
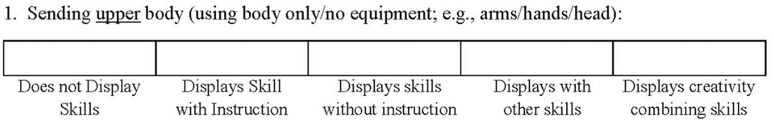
Example item rating scale from Pre-PLAy.

The Pre-PLAy tool was used to assess PL. Research staff trained ECEs on the Pre-PLAy tool in a 45 min session at the center. Training consisted of a review of the tool, and asking ECEs to complete the tool by selecting a child in their classroom and reflecting on their experience with them. Pre-PLAy is comprised of 19 items: 10 items that assess simple skill movement competencies (e.g., sending, transporting), 4 items that assess co-ordinated movements, 4 items that assess motivation and enjoyment, and 1 global item assessing overall PL (see Table 1). For movement competencies, ECEs are asked to rate the skill level for the child considering children of a similar age. This is to ensure that developmentally appropriate comparisons are made. Responses are measured on a modified visual analog scale. ECEs are first asked to select the skill level of the child from the following options: does not display skill, displays skills with instruction, displays skill without instruction, displays with other skills, and creatively displays skills. A rubric is included to describe each skill level in more detail. These are displayed as horizontal connected boxes (see Figure 1 for an example of one item from the scale). Once the level of skill is selected, ECEs are asked to treat the box as a visual analog scale and draw a line vertically in the box to indicate the child's proficiency within the skill level. The further to the right a line placed, the higher the level of PL. The bar was 15 cm long and a ruler was used to measure where the ECE drew the line and determine the child's score for the item from 0 to 15. Co-ordinated movement responses are scored on a 4-point adjectival scale from never to always. Motivation and enjoyment items instruct ECEs to rate their agreement with statements on a 5-point adjectival scale. The final item asks ECEs to rate the child's overall PL using a visual analog scale that is 15 cm long. The Pre-PLAy score was calculated by summing all items in the tool. Scale scores were computed by summing all items in a scale. Item 17 (“When the opportunity to participate in new active games and play that use a variety of movement competencies, the child seems *cautious/hesitant*.”) is reverse scored.

**Table 1:** Items of the preschool physical literacy assessment.

**Domain**	**Items**
Movement competencies	1. Sending upper body (using body only/no equipment; e.g., arms/hands/head): 2. Sending lower body (using body only/no equipment; e.g., legs/feet): 3. Sending with equipment (e.g., bat, stick): 4. Receiving upper body (using body only; e.g. catching with hands/arms): 5. Receiving lower body (using body only; e.g. stopping an object with feet): 6. Receiving with equipment (e.g., glove, stick): 7. Transporting upright (run/hop/jump/skip): 8. Transporting prone (rolling/tumbling): 9. Body control stationary (e.g., maintaining balance while putting on shoes): 10. Body control moving (e.g., is able to maintain balance when moving to catch a ball):
Coordinated movements	11. Uses a variety of moving vehicles (e.g., tricycle; pedal car; scooter) outside during play. 12. Uses playground equipment (e.g., climbing apparatus; slide). 13. Can move inside the classroom without bumping into objects or people who are NOT moving. 14. Can move inside the classroom without bumping into moving objects or people.
Motivation and enjoyment	15. When given the choice, this child will usually choose active games/play that use movement competencies (e.g., jumping, throwing, kicking, etc.) instead of more sedentary activities (e.g., playing in the sandbox, building blocks, coloring). 16. When participating in active games and play that use a variety of different movement competencies, the child often seems confident in his/her abilities. 17. When the opportunity to participate in new active games and play that use a variety of movement competencies, the child seems cautious/hesitant. 18. When participating in active games and play that use a variety of different movement competencies, the child seems to enjoy the experiences.
Overall physical literacy	19. Overall, when thinking about this child's physical literacy (combined movement skills, coordinated action, motivation, and enjoyment), how would you rate this child compared to other children the same age?

#### Physical activity

Objective PA was measured using ActiGraph GT3XP hip-worn accelerometers. ECEs were instructed to have the children wear the accelerometer for the entire time the child was at the day care center, including nap times. Accelerometers were worn at the center for up to 2-weeks (weekdays only). This allowed children who attended the center part time the opportunity to meet the minimum wear time criteria. ECEs recorded the time the child's monitor was put on in the morning, taken off when they left, and other instances when the belt was removed. Nap time was also recorded. Minimum wear time was defined as 3 days of wear time. In order for a day to be counted, the child needed for have worn the accelerometer for at least 4 h of wear time. The 4 h wear time criteria per day meant each day included pre and post nap time programming. Sixty minutes of consecutive zeros was the cut off for a time period to be considered a non-wear period, however, no participant met this cut off. The accelerometer data was cleaned and analyzed using ActiLife Version 6.13.3. Pate, Almeida, McIver, Pfeiffer, and Dowda (16) cut-points were used to define total activity, and moderate to vigorous physical activity (MVPA). We examined minutes of total activity per hour (light, moderate, and vigorous) and minutes of MVPA to define PA.

#### Gross motor skills

The Peabody Developmental Motor Scale [PDMS-2; (17)] was used to assess gross motor skills. The PDMS-2 includes three subscales specifically designed to assess gross motor skills in children aged 1–5 years old: Stationary, Locomotion, and Object Manipulation. Each PDMS-2 assessment was completed by two trained research staff. The assessment took place within 2 weeks of when ECEs completed the first Pre-PLAy for the child. Each child was assessed individually and the assessments took place in areas away from the classroom where distractions were limited (gyms, separate rooms, hallways/corridors). One researcher administered the test (provided instruction and demonstration) and the other scored each item. Scoring was discussed at the end of each assessment and agreed upon by both research staff.

### Procedure

Participating children were matched with two ECEs in each class to assess inter-rater reliability. In classes with three ECEs, random allocation was used to match children to two ECEs. Re-allocation following randomization was required for 8 children to allow ECEs to be matched with children they would have more opportunity to observe due to the structure of the classroom. ECEs were asked to observe children for 2-weeks prior to completing the P-PLAY tool. A second 2-week observation began the day after the first Pre-PLAy tool was completed. This allowed us to test the 2-week test re-test reliability of the tool. Research staff attended the center and completed the PDMS-2 for each child within 2-weeks of the first Pre-PLAy. Research staff completing the PDMS-2 were blinded to Pre-PLAy scores, and ECEs were blinded to the PDMS-2 when completing the P-PLAY.

### Analysis

To assess initial Pre-PLAy item results, test-retest agreement, and validity analysis, a Pre-PLAy from each child was randomly selected when there were two valid scores. Inter-rater and test retest reliability were assessed using a two-way random, consistency, single-measure intraclass correlation (ICC). Criteria outlined by (17) were used to evaluate inter-rater and intra-rater reliability: poor agreement (below 0.4), fair agreement (0.40–0.59), good agreement (0.60–0.74), and excellent agreement (>0.75).

Nesting of the sample occurred at two levels. First, children were nested in classrooms, which were nested in centers. The design effect was calculated using the ICC and cluster size to assess the impact of nesting on the data. A design effect greater than or equal to 2 requires analysis that addresses the nested nature of the data. Minutes of MVPA had a design effects of 3.03. Mixed effects models were employed using R package nlme version 3.1–117 (18) to assess the ability of Pre-PLAy to predict PA. Models were run with classroom nested within center included as a random effect. To probe interaction effects, Preacher, Curran, and Bauer's (19) simple slopes calculator was used.

## Results

### Description of sample

Four children were excluded because they did not have a completed Pre-PLAy or were missing both the PDMS-2 and accelerometer data. This resulted in a sample of 74 children (51% female). Table 2 includes detailed sample characteristics. Average wear time for accelerometers was 7 days across the sample. There were no gender differences in wear time. Boys engaged in more PA than females across all measures. ECEs also rated boys significantly higher on the Pre-PLAy. In contrast, there were no gender differences on the PDMS-2.

**Table 2:** Sample characteristics and differences across sex.

	**Males (*****n*** = **36)**				**Females (*****n*** = **38)**				
	**Mean**	**SD**	**Min**	**Max**	**Mean**	**SD**	**Min**	**Max**	
**DEMOGRAPHIC**									
Age in months	37.36	6.90	27.00	48.00	34.68	8.34	19.00	49.00	n.s.
**PDMS-2 (% Rank)**	**Male (*****n*** = **34)**				**Female (*****n*** = **36)**				
Gross motor	55.24	21.08	4.00	84.00	59.58	21.52	4.00	92.00	n.s.
Stationary	57.09	26.26	9.00	98.00	54.56	24.13	5.00	95.00	n.s.
Locomotion	54.44	22.41	2.00	91.00	58.75	21.45	9.00	98.00	n.s.
Object manipulation	48.15	22.53	9.00	84.00	56.83	22.56	16.00	95.00	n.s.
**Physical activity**	**(*****n*** = **29)**				**(*****n*** = **34)**				
Valid days	7.21	2.32	3.00	10.00	6.97	2.34	3.00	10.00	n.s.
Minutes of MVPA per hour	7.87	1.91	4.51	12.66	6.32	1.62	3.31	10.73	*t_(_*_59)_ = 3.56, *p* = 0.001
Minutes of activity per hour	22.85	2.75	18.42	28.22	20.60	3.14	11.67	27.49	*t_(_*_59)_ = 3.02, *p* = 0.01

*SD, standard deviation; PDMS-2, Peabody Developmental Motor Scale; MVPA, moderate to vigorous physical activity*.

### Pre-PLAy item results

Absences of ECEs and children resulted in three ECEs unable to complete Pre-PLAys on seven children at time 1. Item level missing data was present for 18 children. Item 12 (Uses playground equipment) had a high proportion of missing data (16%) due to the limited availability of playground equipment in some centers: three of the five centers did not have outdoor playground equipment. In those centers, some ECEs felt they could complete the item based on how children played in the natural environment of the playground (e.g., climbing on tree stumps), whereas others did not feel they were able to complete this item. Item 19 was printed on the backside of the last page of the tool. As a result, the item was missed by some ECEs when completing the tool (11%). Due to the high proportion of missing data on these items, the Pre-PLAy total score excluded these items in the current analysis.

Item level distributions showed observed scores were similar to potential scores, indicating ECEs used most or all of the response options when observing children. One exception is that no child was rated over 12 in the movement competencies item. A rating of above 12 would reflect an exceptional level of skill in the area (see Table 3).

**Table 3:** Preschool physical literacy assessment item results.

						**Observed**		**Potential**	
**Item**	**n**	**% missing**	**mean**	**SD**	**median**	**min**	**max**	**min**	**max**
**FEMALE**									
Sending upper body	38	0.0	7.16	2.14	7.55	3.00	10.90	0	15
Sending lower body	38	0.0	6.95	2.19	7.55	3.00	10.10	0	15
Sending with equipment	38	0.0	5.51	2.35	5.55	1.20	10.30	0	15
Receiving upper body	37	2.6	5.99	2.22	6.20	0.90	9.10	0	15
Receiving lower body	38	0.0	5.62	2.11	5.75	1.60	9.70	0	15
Receiving with equipment	38	0.0	4.83	2.12	5.45	1.20	8.80	0	15
Transporting upright	38	0.0	6.98	2.16	7.30	3.00	9.80	0	15
Transporting prone	38	0.0	6.47	2.10	6.90	2.00	9.30	0	15
Body control: stationary	38	0.0	5.64	2.35	5.90	1.30	9.90	0	15
Body control: moving	38	0.0	6.07	2.20	6.30	2.10	10.70	0	15
Uses a variety of moving vehicles	38	0.0	2.61	0.92	2.00	1.00	4.00	1	4
Uses playground equipment	32	15.8	2.56	0.72	2.50	1.00	4.00	1	4
Moves inside the classroom without bumping into objects or people who are NOT moving.	38	0.0	3.11	0.73	3.00	2.00	4.00	1	4
Moves inside the classroom without bumping into objects or people who are moving	38	0.0	2.97	0.75	3.00	2.00	4.00	1	4
Child will usually choose active games/play that use movement competencie	38	0.0	3.24	0.94	3.00	2.00	5.00	1	5
When participating in active games and play, the child often seems confident	38	0.0	3.47	0.86	4.00	2.00	5.00	1	5
When the opportunity to participate in new active games and play, the child seems cautious/hesitant.	38	0.0	2.87	0.96	3.00	1.00	5.00	1	5
When participating in active games and play, the child seems to enjoy the experiences	38	0.0	3.95	0.73	4.00	2.00	5.00	1	5
Overall child's physical literacy	34	10.5	8.00	2.27	7.95	2.90	12.80	0	15
**MALE**									
Sending upper body	36	0.0	8.14	1.97	8.00	3.10	11.90	0	15
Sending lower body	36	0.0	8.16	1.89	8.60	3.10	10.80	0	15
Sending with equipment	36	0.0	7.13	2.35	7.55	0.10	10.60	0	15
Receiving upper body	36	0.0	6.84	1.81	6.90	2.90	11.00	0	15
Receiving lower body	36	0.0	6.26	2.35	6.60	0.50	10.80	0	15
Receiving with equipment	36	0.0	5.23	2.17	5.50	0.10	8.90	0	15
Transporting upright	35	2.8	8.29	1.66	8.20	4.30	11.40	0	15
Transporting prone	35	2.8	7.75	2.34	8.20	1.40	11.60	0	15
Body control: stationary	36	0.0	6.53	2.17	6.70	0.20	9.40	0	15
Body control: moving	36	0.0	6.34	2.58	6.80	0.20	10.90	0	15
Uses a variety of moving vehicles	36	0.0	3.06	0.71	3.00	2.00	4.00	1	4
Uses playground equipment	34	5.6	3.15	0.66	3.00	2.00	4.00	1	4
Moves inside the classroom without bumping into objects or people who are NOT moving.	36	0.0	3.14	0.59	3.00	2.00	4.00	1	4
Moves inside the classroom without bumping into objects or people who are moving	36	0.0	2.83	0.65	3.00	2.00	4.00	1	4
Child will usually choose active games/play that use movement competencie	36	0.0	3.72	1.06	4.00	1.00	5.00	1	5
When participating in active games and play, the child often seems confident	36	0.0	3.78	1.02	4.00	1.00	5.00	1	5
When the opportunity to participate in new active games and play, the child seems cautious/hesitant.	36	0.0	3.47	1.00	3.50	2.00	5.00	1	5
When participating in active games and play, the child seems to enjoy the experiences	36	0.0	4.06	0.71	4.00	2.00	5.00	1	5
Overall child's physical literacy	32	11.1	8.80	2.38	9.25	1.40	12.60	0	15

*SD, standard deviation*.

### Reliability

#### Internal consistency

Internal consistency, corrected item-total correlations, and inter-item correlations were assessed to examine the degree that items on each scale appeared to measure a consistent construct. Correlations for sending items (upper and lower body) and receiving items (upper and lower body) were near or above 0.90. Alphas for the movement competencies, and motivation and enjoyment scale showed good internal consistency (0.941 and 0.841). In contrast, the alpha for coordinated movements was poor (0.623). Item 11 “Uses a variety of moving vehicles” showed poor item-total and inter-item correlations (<0.25). Deleting this item would increase alpha to 0.901.

#### Inter-rater and test-retest agreement

Forty-nine children (66%) had valid Pre-PLAy scores from two ECEs at time 1 and were included in the inter-rater reliability analysis. Sixty-five children (88%) had valid Pre-PLAy scores from an ECE 2 weeks apart. Detailed results for inter-rater and test retest agreement are shown in Table 4. Rater agreement varied by sex, for females, agreement between ECEs ranged from fair to excellent on most items. The exception were the coordinated movements scale score and items which showed poor agreement. ECE's ratings were more divergent for males, with agreement on eight items below the acceptable range. Of the remaining items for boys, only the two sending items showed good agreement. Test-retest agreement across a 2-week period for items was fair to good across all items for both females and males. Again, the co-ordinated movements scale had the lowest reliability value for both females and males.

**Table 4:** Agreement between early childhood educators and across time.

		**Inter-rater ICC**		**Intra-rater ICC**	
	**Item**	**Male (*n* = 23)**	**Female (*n* = 26)**	**Male (*n* = 34)**	**Female (*n* = 33)**
1	Sending upper body	0.71	0.58	0.72	0.52
2	Sending lower body	0.78	0.59	0.78	0.55
3	Sending with equipment	0.43	0.62	0.73	0.59
4	Receiving upper body	0.33	0.76	0.61	0.59
5	Receiving lower body	0.21	0.57	0.54	0.72
6	Receiving with equipment	0.44	0.51	0.59	0.70
7	Transporting upright	0.17	0.75	0.71	0.53
8	Transporting prone	−0.08	0.62	0.57	0.50
9	Body control: stationary	0.42	0.64	0.55	0.67
10	Body control: moving	0.09	0.62	0.41	0.64
11	Uses moving vehicles	0.44	0.22	0.72	0.63
13	Moves inside the classroom without bumping into objects or people who are NOT moving.	0.17	0.24	0.51	0.49
14	Moves inside the classroom without bumping into objects or people who are moving	0.33	0.38	0.46	0.59
15	Child will usually choose active games/play that use movement competencie	0.51	0.71	0.74	0.73
16	When participating in active games and play, the child often seems confident	0.49	0.57	0.53	0.57
17	When the opportunity to participate in new active games and play, the child seems cautious/hesitant.	0.19	0.79	0.47	0.50
18	When participating in active games and play, the child seems to enjoy the experiences	0.57	0.65	0.48	0.51
	Pre-PLAy Total Score	0.42	0.66	0.79	0.73
	Movement competencies	0.33	0.64	0.74	0.70
	Co-ordinated movements	0.33	0.28	0.58	0.58
	Motivation and enjoyment	0.55	0.79	0.73	0.76
ICC, intraclass correlation; Pre-PLAy, Preschool Physical Literacy Assessment					

#### Convergent validity

The relationships between the Pre-PLAy tool and PDMS-2 varied across gender. For females, correlations were low to moderate range between Pre-PLAy total with the total gross motor scale of the PDMS-2, stationary, and locomotion subscales. The same pattern of results was found for the movement competencies subscale of the Pre-PLAy. No significant correlations were found with the coordinated movements and motivation and enjoyment domains. No significant associations between the Pre-PLAy and PDMS-2 were found for males (see Table 5).

**Table 5:** Spearman's Rho correlations with peabody developmental motor scales.

	**Males (*****n*** = **33)**				**Females (*****n*** = **39)**			
	**Gross motor % rank**	**Stationary % rank**	**Locomotion % rank**	**Object manipulation % rank**	**Gross motor % rank**	**Stationary % rank**	**Locomotion % rank**	**Object manipulation % rank**
Pre-PLAy total	−0.118	−0.243	0.238	0.225	0.388^*^	0.483^*^^*^	0.358^*^	0.095
Movement competencies	−0.122	−0.251	0.257	0.233	0.369^*^	0.483^*^^*^	0.357^*^	0.048
Co-ordinated movements	0.112	−0.279	−0.096	−0.056	0.288^+^	0.338	0.148	0.187
Motivation and enjoyment	−0.005	−0.037	0.123	0.161	0.235	0.268	0.176	0.144

*Pre-PLAy, Preschool Physical Literacy Assessment*.

* p < 0.05,

+ *p < 0.10; Boldface indicates a statistically significant effect*.

**Figure 2:**
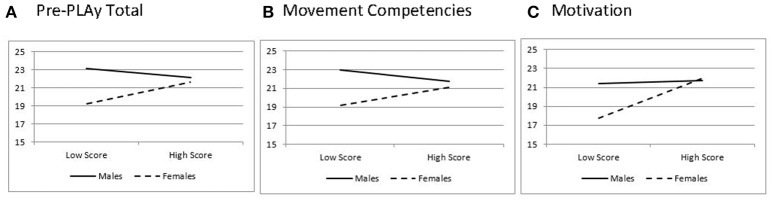
Interaction for minutes of physical activity per hour for **(A)** Pre-PLAy total, **(B)** movement competencies, and **(C)** motivation.

**Figure 3:**
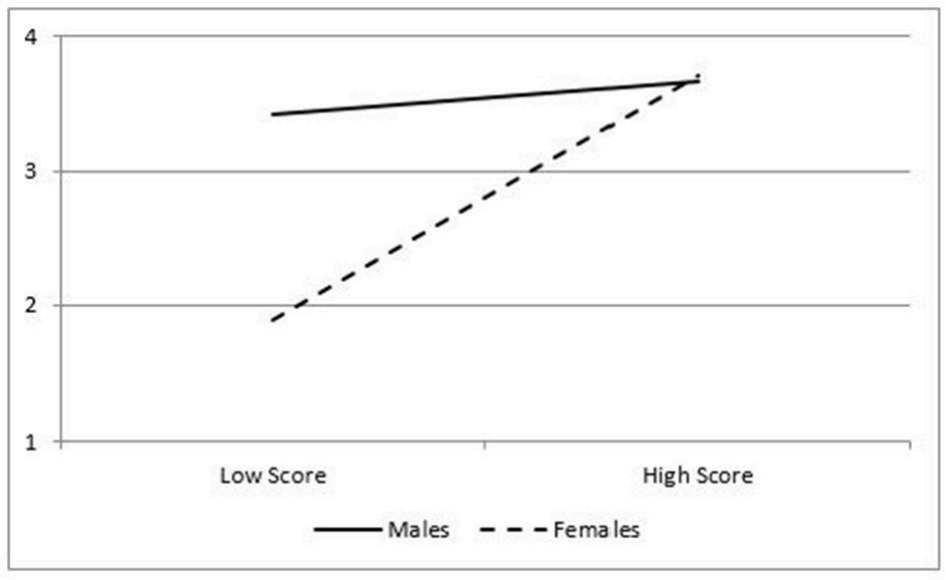
Interaction for minutes of moderate to vigorous activity per hour and motivation subscale.

The relationship between the Pre-PLAy and PA was moderated by gender. Gender moderated the ability of the Pre-PLAy to predict minutes of activity per hour, except coordinated movements (see Table 6). Gender also moderated the relationship between motivation and minutes of MVPA per hour. Simple slopes analyses were conducted to probe the interactions. Results for males and females were plotted at 1 standard deviation (SD) below and above the mean in Figures 2, 3. Interactions showed the Pre-PLAy predicted minutes per hour of PA for girls (*B* = 0.06, SE = 0.03, *z* = 2.27, *p* = 0. 02) and was not significant for boys (*B* = −0.03, SE = 0.03, *z* = −0.85, *p* = 0.40). The movement competency domain of the Pre-PLAy approached significance for females (*B* = 0.06, SE = 0.03, *z* = 1.76, *p* = 0.08), and the motivation domain significantly predicted Pre-PLAy scores for females (*B* = 0.73, SE = 0.18, *z* = 4.00, *p* = 0.0001). Neither domain significantly predicted Pre-PLAy in males (*B* = −0.04, SE = 0.04, z = −0.99, *p* = *0.3*2; *B* = 0.05, SE = 0.18, *z* = 0.26, *p* = *0.8*0). A similar pattern was found between motivation and MVPA with significant results for females (*B* = 0.32, SE = 0.09, *z* = 3.39, *p* = 0.0007) and non-significant results for males (*B* = 0.04, SE = 0.09 *z* = 0.48, *p* = 0.63).

**Table 6:** Mixed effects model of preschool physical literacy assessment on physical activity.

	**Activity (L**+**M**+**V)**		**MVPA**	
	**Estimate**	**s.e**.	**Estimate**	**s.e**.
Pre-PLAy total	−0.027	0.032	0.003	0.017
Sex	−2.170^**^	0.777	−0.882^**^	0.410
Pre-PLAy *sex	0.091^**^	0.042	0.015	0.022
Movement competencies	−0.037	0.037	0.001	0.019
Sex	−2.253^**^	0.784	−0.930^**^	0.411
Movement competencies ^*^sex	0.095^**^	0.049	0.010	0.025
Coordinated movements	−0.150	0.336	−0.005	0.170
Sex	−2.044^**^	0.755	−0.938^**^	0.384
Coordinated movements ^*^sex	0.716	0.439	0.219	0.218
Motivation	0.047	0.183	0.043	0.090
Sex	−1.727^**^	0.725	−0.734^**^	0.372
Motivation ^*^sex	0.686^**^	0.259	0.277^**^	0.132

*Pre-PLAy, Preschool Physical Literacy Assessment; L+M+V, Light, Moderate, and Vigorous Physical Activity; MVPA, moderate to vigorous physical activity*.

*All models are adjusted for age*,

* *p < 0.05*,

** *p < 0.01; Boldface indicates a statistically significant effect*.

## Discussion

This is the first study that has tested a PL assessment instrument in preschool children. Results of the study suggest promise for the Pre-PLAy tool, but also areas in need of modification. In terms of promise, for females, the tool demonstrated adequate reliability, and there was evidence of validity in agreement with a standardized measure of gross motor skills and objectively measured PA. In contrast, results for males were poor, with many items demonstrating poor reliability, and no significant agreement with gross motor skills and PA.

The discrepant results for boys and girls indicate a potential sex bias in ECE ratings. While there is a dearth of research on this in the early years setting, prior research with teachers ratings of school-aged children have shown sex biases related to motor competence. For example, Hay and Donnelly (20) found teachers overestimated boys motor abilities and underestimated girls. There is also evidence that the sex of the teacher can impact ratings of child development (21), however the reason for these sex differences has not been examined. Therefore, our results might suggest ECEs hold different beliefs regarding the motor skills of males and females in this developmental period. These beliefs might vary based on cultural differences, years in the field, and whether the ECE is male or female. In the current study, all ECEs were female so rater bias attributable to sex of the evaluator is not an issue. Unfortunately, we did not collect additional information about years of training or experience of ECEs in our sample. The beliefs and perceptions of ECEs about motor skill is an important, and understudied area of research.

Another factor that can help explain the lack of agreement for males is the presence of disruptive behaviors. Rivard et al. (22) found the presence of disruptive behaviors suppressed teacher concerns about motor skill in school aged children. Disruptive behaviors are most prevalent, and are in fact normative during the preschool years, and higher among males compared to females (23, 24). Therefore, ECEs might be less accurate in assessing a child's PL if the child engages in more disruptive behaviors, and this is more likely to occur among male preschool children.

These results suggest the need for further training with ECEs to understand and address sex differences in Pre-PLAy ratings. Training on the tool focused on ensuring ECEs understood the instrument, and discussion around different opportunities to observe children during regular programming to complete the tool. Ensuring there are opportunities to use the tool with both male and female children, and to discuss and address any discrepancies will be an important area of development for training on the tool.

Item level results suggest additional revisions could improve the tool. For example, when assessing movement competencies, ECEs did not rate any child in the creatively combines skills category. If this is replicated in future studies with larger samples, it could be beneficial to remove this category. The high proportion of missing data on the uses playground equipment item suggests modifying the tool to either revise the item to include other active play equipment in centers or making the item optional in scoring the tool would be useful. The high correlations between upper and lower body skills suggests ECEs did not distinguish between the two items. Specific training focusing on upper and lower body sending and receiving might help ECEs better distinguish between these items, though this needs to be tested in future research.

Internal consistency, corrected item-total, and inter-item correlations were supportive of the multidimensional structure of the Pre-PLAy. Most items fit well on their respective scales with the exception of item 11 (uses a variety of moving vehicles), which item did not fit with the other coordinated movement items based on item total correlations and inter-item correlations. Removal of this item would also improve the alpha of the scale. Results suggest either modifying or removal of the coordinated movements scale might be warranted. The coordinated movements scale demonstrated inadequate inter-rater agreement for males and females and the lowest intra-rater agreement of all the Pre-PLAy scales. It also did not appear to contribute to the validity of the tool with no significant relationship with the PDMS-2 or PA. However, considering limitations of the current study (including potential gender bias in the study and the small sample size), further testing of the scale in a larger sample might be warranted before removal.

We conducted a real-world test of the Pre-PLAy tool with ECEs completing the Pre-PLAy tool alongside their regular programming and responsibilities. Results of the study are important for both future work in preschool PL, and also for research in the early years more broadly. While there is a body of research on a number of observational instruments for teachers and school-aged children, there has been little examination of similar types of tool for ECEs and preschool children. Results suggest further research on the Pre-PLAy tool is warranted.

## Ethics statement

This study was carried out in accordance with the recommendations of Hamilton Integrated Research Ethics Board with written informed consent from all parents of participating children. All parents of participating children gave written informed consent in accordance with the Declaration of Helsinki.

## Author contributions

JC conceived of the study design with input from HC and contributed to the writing and editing of the manuscript; HC analyzed the data and contributed to the writing of the manuscript; MJ aided in collecting and analyzing the data, and revised the manuscript; DM, DD, and DK all reviewed and contributed to the manuscript. All authors participated in the development of the Pre-PLAy tool.

### Conflict of interest statement

The authors declare that the research was conducted in the absence of any commercial or financial relationships that could be construed as a potential conflict of interest.
